# TGF-β1-overexpressing mesenchymal stem cells reciprocally regulate Th17/Treg cells by regulating the expression of IFN-γ

**DOI:** 10.1515/biol-2021-0118

**Published:** 2021-11-02

**Authors:** Ruixue Li, Renyong Wang, Shijie Zhong, Farhan Asghar, Tiehan Li, Lei Zhu, Hong Zhu

**Affiliations:** Department of Hepatobiliary and Pancreatic Surgery, The Second Affiliated Hospital of Kunming Medical University, Kunming, Yunnan Province, 650101, China

**Keywords:** TGF-β1, MSC, Th17, Treg, immunosuppression

## Abstract

Transforming growth factor (TGF)-β1 and mesenchymal stromal cells (MSCs) are two effective immunosuppressive agents for organ transplantation technology. This study aims to explore the molecular mechanism of TGF-β1-overexpressed MSCs on T cell immunosuppression. To achieve that, BM-MSCs were isolated from canine bone marrow, and their osteogenic differentiation and surface markers were detected. The TGF-β1 gene was transferred into lentivirus and modified MSCs (TGF-β1/MSCs) by lentivirus transfection. Furthermore, TGF-β1/MSCs were co-cultured with T cells to investigate their effect on differentiation and immune regulation. Results showed that TGF-β1/MSCs significantly downregulated the proportion of CD4^+^ CD8^+^ T cells in lymphocytes and significantly upregulated the proportion of CD4^+^ CD25^+^ T cells. Moreover, TGF-β1/MSCs significantly upregulated the expression of IL-10 in CD4^+^ T cells and downregulated the expression of IL-17A, IL-21, and IL-22. Meanwhile, interferon-γ (IFN-γ) neutralizing antibody blocked the effects of TGF-β1/MSCs on the differentiation inhibition of Th17. Overall, our results confirm the strong immunosuppressive effect of TGF-β1/MSCs *in vitro* and demonstrate that IFN-γ mediates the immunosuppressive effect of TGF-β1/MSC.

## 1 Introduction

With the continuous development and progress of modern medicine, the transplantation of allogeneic tissues and organs has become necessary for various diseases. However, the acute or chronic rejection that often occurs after allotransplantation seriously restricts the further development and application of transplantation technology. At present, immunosuppressants are mainly used to inhibit rejection after transplantation to prolong the survival time of grafts or transplant recipients; however, recipients need to take immunosuppressant drugs for life. Moreover, it may produce various side effects and increase the risk of opportunistic infection, causing a significant burden to patients, which seriously limits the scope of its clinical application. Therefore, it is a crucial problem to be solved urgently in transplantation technology to find a method that can induce allograft immune tolerance, prolong graft survival time, have low side effects, and high safety factors.

Bone marrow mesenchymal stem cells (MSCs) belong to nonterminally differentiated cells, which have the characteristics of mesenchymal cells and the potential of stem cell self-renewal and multidirectional differentiation. Animal experiments and preliminary clinical transplantation studies have found that MSCs play an essential role in supporting stem cell implantation and regulating transplantation immunity. After the infusion of MSCs to patients receiving allogeneic skin transplantation, it was found that MSCs could significantly prolong the survival time of allogeneic skin transplantation [1]. A study on the use of mesenchymal stem cells in cartilage regeneration in osteoarthritis has shown that MSCs have special low immunogenicity and immunomodulatory ability [2]. Another study of 47 patients with intractable graft-versus-host disease showed that bone marrow MSCs isolated from third parties effectively treated intractable graft-versus-host disease [3]. The unique immunological characteristics of MSCs enable them to survive for a long time in an allogeneic or even xenogeneic body. After transplantation, MSCs still maintain the potential of multidirectional differentiation but its antigenicity is not increased, which plays a vital role in studying allogeneic or mismatched transplantation. Preliminary studies have shown that autologous or allogeneic bone marrow-derived MSC transplantation does not cause significant adverse reactions. The low immunogenicity of MSCs enables them to evade immune recognition in immune response and gain the ability to survive in the receptor. Besides, MSCs can inhibit T cell proliferation through direct cell-to-cell contact and the indirect effect of cytokines secreted by MSC; moreover, the inhibitory effect of MSCs is dose-dependent, that is, the greater the number of MSCs, the stronger the inhibitory effect [4].

Transforming growth factor β (TGF-β) is a widely used immunosuppressive agent regulating proliferation, differentiation, and biological function of many kinds of immunoreactive cells [5]. Its role in immunity has attracted wide attention, such as inducing B cells to secrete IgA [6]. Particularly, it can stimulate immature CD4^+^ T cells to differentiate into Foxp3 Treg and inhibit the activation and proliferation of effector T cells [7]. TGF-β leads to cell inactivation and plays a negative regulatory role, thus maintaining immune tolerance [8,9]. Johnston et al. proved that TGF-β upregulated regulatory T cells (Treg), induce immune tolerance, and prolong grafts’ survival time [10]. The activity of TGF-β1 and MSCs can regulate rejection after transplantation.

Based on the above, this study explored the molecular mechanism of TGF-β1 modification of MSCs on T cell immunosuppression. Compared with the blank control group, TGF-β1/MSCs significantly downregulated the proportion of CD4^+^ CD8^+^ T cells in lymphocytes and significantly upregulated the proportion of CD4^+^ CD25^+^ T cells in lymphocytes. In addition, we also found that TGF-β1/MSCs significantly upregulated the expression of IL-10 in CD4^+^ T cells and downregulated the expression of IL-17A, IL-21, and IL-22, which explained the immunosuppressive effect of TGF-β1/MSC. Meanwhile, IFN-γ neutralizing antibody blocked the effects of TGF-β1/MSCs on the differentiation inhibition of Th17. Overall, our results confirmed the strong immunosuppressive effect of TGF-β1/MSCs *in vitro* and found that IFN-γ mediated the immunosuppressive effect of TGF-β1/MSC.

## 2 Material and methods

### 2.1 Preparation of canine bone marrow mesenchymal stem cells and gene transduction

Under an aseptic operation, the experimental dogs were anesthetized and punctured at the left or right skeletal crest with a bone marrow puncture needle. First, 3 mL of the bone marrow fluid was extracted into a preprepared 15 mL centrifuge tube containing anticoagulants, and then 3 mL of PBS buffer containing double antibodies was diluted to make 6 mL bone marrow dilution. Clumps were removed by filtering through a 70 μm cell strainer (Thermo-Fisher Scientific). Cells were then centrifuged at 400×*g* for 5 min and then resuspended in the complete culture medium of stem cells. The cell suspension was inoculated in a 60 mm Petri dish and cultured at 37°C in a 5% CO_2_ incubator for 24 h. Lentiviral vectors for canine TGF-β1 gene overexpression were constructed by and purchased from Genscript (Nanjing, China). The cells were transduced with the recombinant virus for 12 h and then cultured in the viral vector-free medium for another 48 h. Subsequently, the MSCs transduced with TGF-β1 were exposed to 5 mg/mL puromycin for 3 days to obtain stable transduction TGF-β1/MSCs. To neutralize the IFN-γ, neutralizing antibodies (NA) for IFN-γ (10 μg/mL) were added to the co-culture; NA to IFN-γ were obtained from R&D Systems (Minneapolis, MN).


**Ethical approval:** The research related to animal use has been complied with all the relevant national regulations and institutional policies for the care and use of animals and was approved by the Animal Ethics Committee of the Second Affiliated Hospital of Kunming Medical University.

### 2.2 Identification of osteogenic differentiation of MSCs

When the cell density reaches 80%, 1/3rd of bone marrow MSCs were digested with trypsin. The cell concentration was adjusted to 3 × 10^4^ cells/mL and seeded in 6-well plates. About 2 mL of growth medium (stem cell complete medium) was added to each well and cultured at 37°C in 5% CO_2_ incubator until the cells were covered with culture plates. After 24 h of induction and differentiation, the growth medium was carefully discarded, and 2 mL of the osteogenic induction medium was added to the experimental group. After alizarin red staining, the experimental group cells were induced to form calcium nodules as far as possible. The induced differentiation solute ion was discarded entirely, washed with PBS, and fixed with 4% formaldehyde solution (2 mL for 30 min); after fixation, it was washed twice with PBS and washed 2–3 times with 1 mL of alizarin red staining solution for 3 min. The staining results were observed under an inverted microscope and photographed.

### 2.3 Flow cytometry cell sorting and analysis

For cell sorting, splenocytes from 7- to 8-week-old BALB/C mice were used to purify CD4^+^, CD8^+^ or CD25^+^ T cells according to the manufacturer’s instructions (BD FACSAria Ⅲ, BD Biosciences, Germany). The purity of T cells was confirmed by flow cytometry. For cell surface staining, the cells were resuspended in PBS (1 × 10^5^ cells/mL). Fluorescence-labeled monoclonal antibodies anti-CD4, anti-CD25, anti-CD34, anti-CD105, anti-CD44, anti-CD29, and anti-CD90 were, respectively, added and incubated in the refrigerator at 4°C for 30 min. All antibodies for flow cytometry were purchased from BD Biosciences. Flow cytometry analysis was performed with a FACS Aria II Cell Sorter (BD Biosciences) and data analysis was carried out using FlowJo software (TreeStar, Ashland, USA).

### 2.4 Western blotting

Proteins were extracted and subjected to Western blot analysis. Equal amounts of proteins (20 μg) were conducted by SDS-PAGE (12%) and transferred to the PVDF membrane (Epizyme, Shanghai, China) by semidry blotting. After blocking with 5% (w/v) non-fat milk powder, membranes were probed with the primary antibody against β-actin (1:5,000 dilution), IL-17 (1:1,000 dilution), Foxp3 (1:1,000 dilution), and RORγt (1:1,000 dilution). All antibodies were purchased from CST (Shanghai, China). The secondary antibodies were HRP conjugated goat anti-rabbit IgG (1:5,000 dilutions, Proteintech, Wuhan, China). Signals were detected by the chemiluminescence procedure (Pierce, Rockford, Illinois, USA) with BioMax films (Kodak).

### 2.5 Real-time quantitative PCR

RNA extraction and quantitative real-time PCR were performed for gene expression analysis. In short, according to the manufacturer’s protocols, the total RNA was extracted from the cell sample with the TRIzol reagent (Invitrogen, Carlsbad, CA, USA). The RNA concentration was measured using a spectrophotometer, and all samples were balanced by reverse transcription with a cDNA synthesis kit (Fermentas, St. Leon-Rot, Germany). Using cDNA as templates, SYBR Green I dye-labeled fluorescence quantitative detection was performed at 95°C for 10 min, followed by 40 cycles of 95°C, 20 s and 60°C, 30 s. Assays were performed in duplicate or triplicate as 20 µL reactions in 96 well plates using the 7,500 Fast Real-Time PCR System (Applied Biosystems, Foster City, CA, USA). Using the 2^−ΔΔCt^ method, GAPDH was used as an internal reference to quantify the expression level of target genes in each experimental group. The primers used were synthesized as follows: GAPDH, 5′-AGA CAG CCG CAT CTT CTT GT-3′ (forward), GAPDH, 5′-TGA TGG CAA CAA TGT CCA CT-3′ (reverse); IL-17, 5′-CTC CAG AAG GCC CTC AGA CTAC-3′ (forward), IL-17, 5′-AGC TTT CCC TCC GCA TTG ACA CAG-3′ (reverse); T-bet, 5′-TCA CTA AGC AAG GAC GGA GAA TG-3′ (forward), T-bet, 5′-ATA AGC GGT TCC CTG GCA TAC-3′ (reverse); Foxp3, 5′-CAA GTT CCA CAA CAT GCG AC-3′ (forward), Foxp3, 5′-ATT GAG TGT CCG CTG CTT CT-3′ (reverse); IL-17F, 5′-ACC AAG GCT GCT CTG TTT CT-3′ (forward), IL-17F, 5′-GGT AAG GAG TGG CAT TTC TA-3′ (reverse); RORγt, 5′-TGG AAG TGG TGC TGG TTA GGA TG-3′ (forward), RORγt, 5′-GGA GTG GGA GAA GTC AAA GAT GGA-3′ (reverse); RORα, 5′-CTA CAT TGA CGG GCA CACC-3′ (forward), RORα, 5′-ACA CAG TTG GGG AAG TCT CG-3′ (reverse).

### 2.6 Quantification of cytokines

After the cells were treated according to the experimental conditions, the culture medium was collected. Then, the culture medium was centrifuged at 4°C for 10 min. The contents of IFN-γ, IL-4, IL-10, IL-21, IL-22, and IL-17A in the supernatant were detected using the ELISA kit (Dakewe, Beijing, China).

### 2.7 Inhibitory effects of TGF-β1/MSCs on T cells

CD4^+^ T and CD8^+^ T cells were isolated from the bone marrow MSCs. All 2.5 × 10^5^ CD4^+^ T cells per well were co-cultured with MSCs (3 × 10^5^) or TGF-β1/MSCs. The proliferation of CD4^+^ T cells was measured after 3, 5, or 7 days using the MTT assay [11]. The percentage of suppressive capacity was calculated by defining the absorption of the control as 100%. All CD8^+^ T cells were co-cultivated with IL-2 (40.0 U/mL) and co-cultured with MSCs or TGF-β1/MSCs. After 3, 5, or 7 days, an LDH assay (Beyotime Biotechnology, Nantong, China) was performed to analyze the cytotoxic lymphocyte (CTL) activity. Then, MDCK cells were added to the effector cells. All cells were cultured under 5% CO_2_ at 37°C for 24 h, and the cell-free supernatant was then retrieved after centrifugation at 500×*g* for 5 min to analyze LDH. Then, the CTL killing activity was calculated as previously described [12].

### 2.8 Statistical analysis

Origin 9.0 for Windows was employed in the data analysis. GraphPad Prism 5 was also adopted for preliminary data analysis. All data were expressed as mean ± SD of individual values. The statistical analysis was determined using Student’s *t*-test. *P* < 0.05 was considered statistically significant.

## 3 Results

### 3.1 Isolation and identification of BM-MSCs

**Figure 1:**
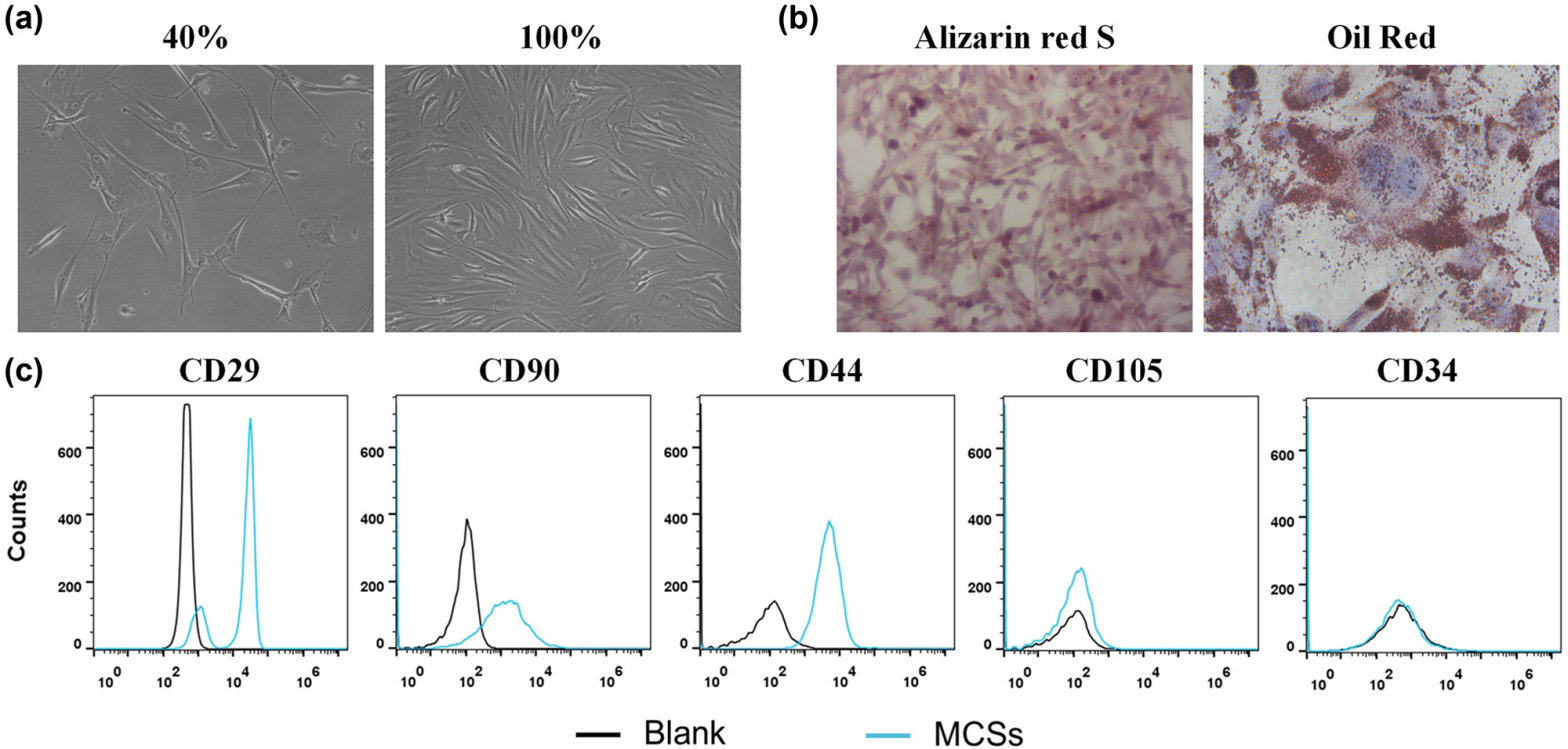
Characterization of BM-MSCs. (a) The basic morphology of BM-MSCs. (b) Identification of osteogenic differentiation ability of BM-MSCs. (c) Phenotype identification of BM-MSCs was conducted by flow cytometry.

The cell suspensions isolated from the bone marrow of the femur and tibia of mice were cultured. The next day, the nonadherent cells and impurities were removed by changing the culture medium. With the growth and proliferation of the cells, the adherent cells gradually became fusiform and showed colony-like growth (Figure 1a). To confirm the stemness of MSCs, the osteogenic and adipogenic differentiation potential were determined. After osteogenic induction and differentiation culture, granular substances could be observed in the cells after about 5–7 days, and their levels increased gradually. After about 3 weeks, a large number of granular substances could be observed in the cells, evenly suspended in the culture medium, and alizarin red staining showed positive results, which proved the osteogenic differentiation ability of the extracted cells. Similarly, oil red staining results also showed many lipid droplets, indicating that the isolated MSCs were functional (Figure 1b). The flow cytometry results showed that CD44, CD90, and CD29 were positively expressed, while CD105 and CD34 were negatively expressed (Figure 1c). These results were consistent with the characteristics of MSCs.

### 3.2 Detection of transfection efficiency

**Figure 2:**
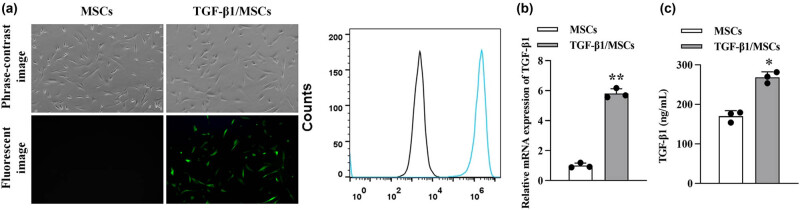
Characterization of TGF-β1/MSCs. (a) MSCs were transduced with lentivirus-GFP and lentivirus-TGF-β1. (b) The transduction efficiency was analyzed using flow cytometry. (c) The mRNA level of TGF-β1 in MSCs and TGF-β1/MSCs. (d) The concentration of TGF-β1 in the supernatants of MSCs and TGF-β1/MSCs. Data are expressed as the mean ± SD. Three independent experiments were analyzed using unpaired Student’s *t*-tests. *P < 0.05; **P < 0.01.

After 96 h of TGF-β1-lentivirus transfection, massive green fluorescence (GFP expression) was observed under a fluorescence microscope in the TGF-β1/MSCs group (Figure 2a), which was also confirmed by flow cytometry (Figure 2b). We further detected the expression of TGF-β1. The results showed that the mRNA level and protein expression of TGF-β1 were significantly increased in TGF- β1/MSCs compared to MSCs.

### 3.3 TGF-β1/MSCs inhibited T lymphocyte proliferation and the regulation of T lymphocyte immune status

**Figure 3:**
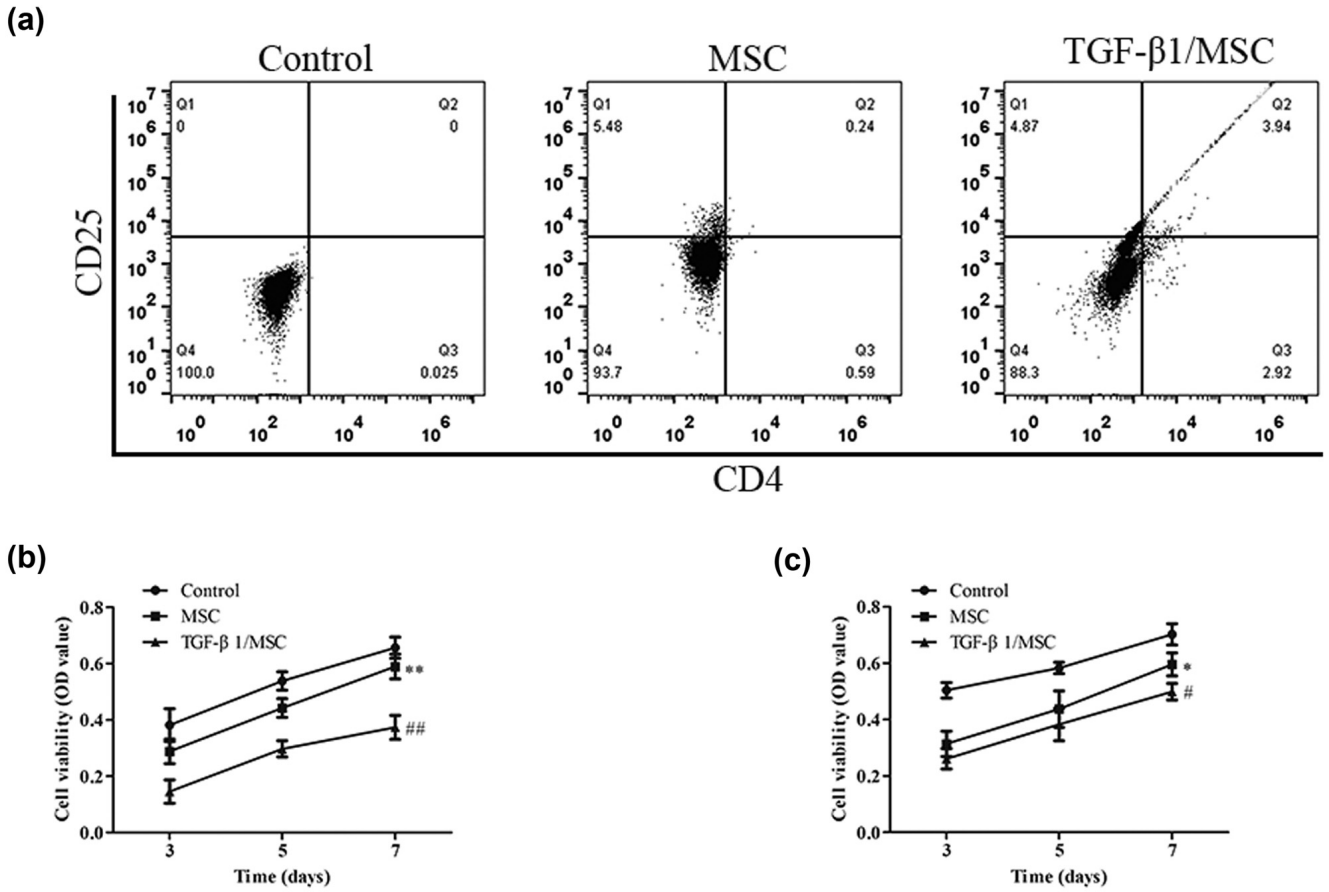
The effect of TGF-β1/MSCs on T cells. (a) T cells were co-cultured with MSCs or TGF-β1/MSCs, and the proportion of CD4^+^ CD25^+^ T cells in lymphocytes was detected by flow cytometry. (b) CD4^+^ T cells were co-cultured with MSCs or TGF-β1/MSCs for 3, 5, or 7 days, and proliferative responses were measured. (c) CD8^+^ T cells were co-cultured with MSCs or TGF-β1/MSCs in the presence of IL-2 after 3, 5, or 7 days. Live cells were collected, and the cytotoxic activity against MDCK cells was assessed. Data are expressed as the mean ± SD. Three independent experiments were analyzed using unpaired Student’s *t*-tests. *P < 0.05; **P < 0.01; MSCs group vs control group. ^#^ *P* < 0.05; ^##^ *P* < 0.01; TGF-β1/MSCs group vs MSCs group.

**Figure 4:**
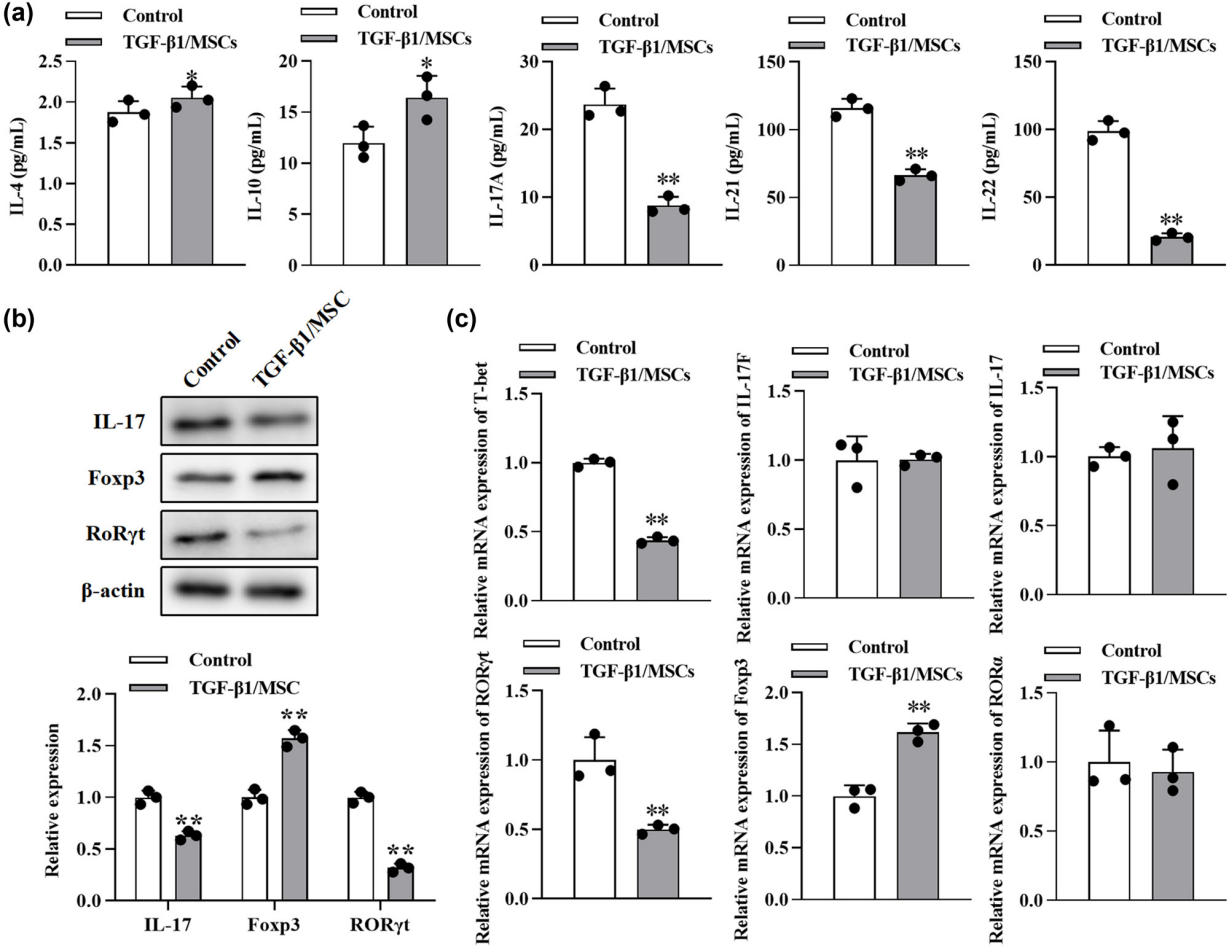
The effect of TGF-β1/MSCs on Th17 differentiation of T cells. (a) After CD4^+^ CD25^−^ T cells were cultured with TGF-β1/MSCs for 3 days in the presence of IL-2, cytokines (IL-4, IL-10, IL-17A, IL-21, and IL-22) were measured by ELISA from the culture supernatant. (b) The expression of IL17, Foxp3, and RORγt in T cells was measured by Western blotting. (c) The mRNA levels of T-bet, IL-17, IL-17F, RORγt, Foxp3, and RORα were detected using RT-PCR. Data are expressed as the mean ± SD. Three independent experiments were analyzed using unpaired Student’s *t*-tests. *P < 0.05; **P < 0.01; TGF-β1/MSCs group vs control group.

T lymphocytes were isolated and purified, and the inhibition of T lymphocyte proliferation and T lymphocyte immune status regulation was observed in MSCs and TGF-β1/MSCs. The proportion of CD4^+^ CD25^+^ T cells in mixed lymphocytes was measured by flow cytometry. As shown in Figure 3a, the proportion of CD4^+^ CD25^+^ T cells increased under the stimulation of MSCs and TGF-β1/MSCs, and the TGF-β1/MSCs group increased more than the MSCs group. To evaluate the effect of TGF-β1/MSCs on allogeneic stimulation, the mixed lymphocyte reaction of CD4^+^ T cells and the CTL activity of CD8^+^ T cells were evaluated. Figure 4b shows that CD4^+^ T cells from the TGF-β1/MSCs group had markedly decreased proliferative functions than those from the MSCs group and control group. Further, the control group showed a significant increase in the allospecific CTL activity (Figure 4c).

### 3.4 TGF-β1/MSCs promoted the generation of Treg cells and suppressed the differentiation to Th17

**Figure A1:**
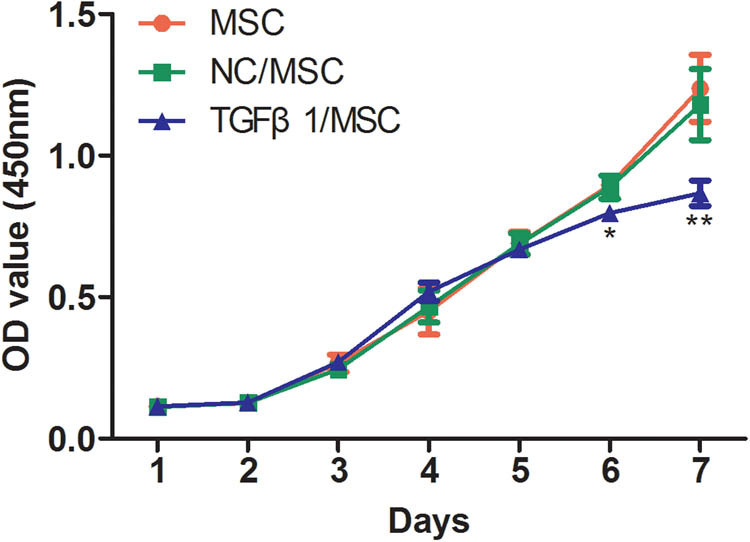
The effects of overexpressing TGFβ1 on the proliferation of MSCs. The growth curves of the cells after transduction for 7 days were evaluated by the CCK-8 assay. *P  <  0.05, **P  <  0.01; TGF-β1/MSCs group vs MSCs group.

We further tested the effects of TGF-β1/MSCs on the differentiation of T cells. In order to exclude the effect of TGF-β1 transfection on the proliferation of MSCs, we conducted the CCK-8 assay. The proliferation of MSCs after overexpression of TGF- β1 was not significantly different from that of the control group at 1–5 days but was inhibited at 5–7 days (Figure A1). Next, CD4^+^ CD25^−^ T cells were co-cultured with TGF-β1/MSCs, and the results showed that the level of IL-10 in the culture supernatant was higher than that in the control group (Figure 4a). However, the levels of IL-17A, IL-21, and IL-22 were lower than those in the control group, and the level of IL-4 was similar to that in the control group. TGF-β1/MSCs could increase the expression of Foxp3 cells but suppressed the expression of IL17 and RORγt in CD4^+^ CD25^−^ T cells (Figure 4b). Furthermore, CD4^+^ CD25^−^ T cells in these co-cultures expressed a higher mRNA level of Foxp3 but lower mRNA level of T-bet and RORγt (Figure 4c). These results revealed that TGF-β1/MSCs co-cultured with CD4^+^ CD25^−^ T cells promoted the generation of Treg cells and suppressed the differentiation to Th17 cells.

### 3.5 TGF-β1/MSCs inhibited Th17 cell differentiation via IFN-γ

**Figure 5:**
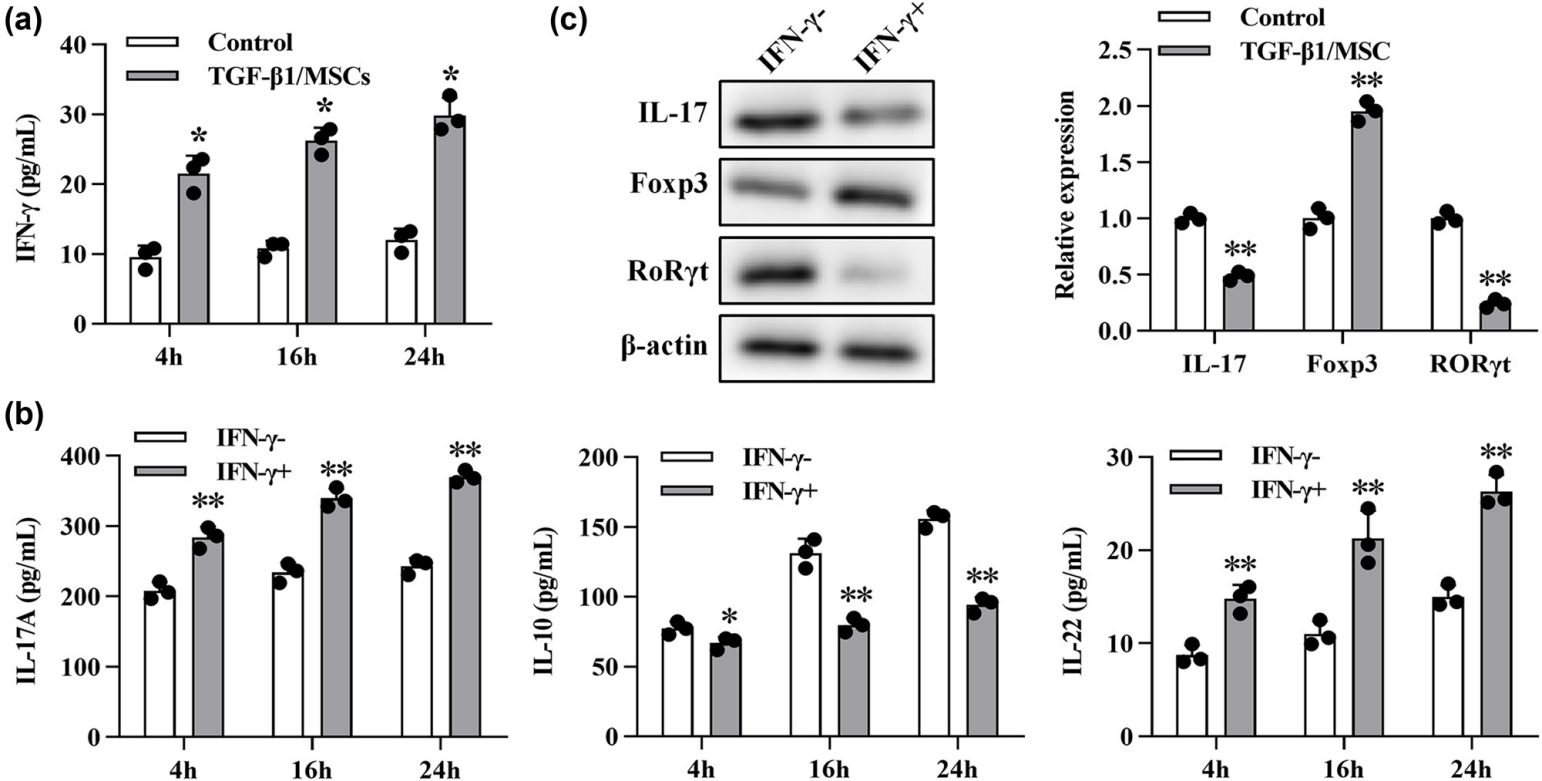
TGF-β1/MSCs regulate Th17 cell differentiation through IFN-γ. (a) CD4^+^ T cells were co-cultured with TGF-β1/MSCs, and the concentration of IFN-γ in the supernatants was measured. *P < 0.05; **P < 0.01; TGF-β1/MSCs group vs control group. (b) Cytokines were measured by ELISA using the culture supernatant in which CD4^+^ T cells were co-cultured with TGF-β1/MSCs in the presence of IFN-γ NA. *P < 0.05; **P < 0.01; IFN-γ + group vs IFN-γ- group. (c) The expression of IL17, Foxp3, and RORγt in T cells was measured by Western blotting in the presence of IFN-γ NA. *P < 0.05; **P < 0.01; TGF-β1/MSCs group vs control group. Data are expressed as the mean ± SD. Three independent experiments were analyzed using unpaired Student’s *t*-tests.

Next, we further explored the intrinsic mechanisms underlying the inhibition of Th17 differentiation. A high level of IFN-γ was observed in the co-culture (Figure 5a), so we conjectured that IFN-γ might be related to the differentiation of Th17. Hence, to neutralize IFN-γ, NA for IFN-γ (10 μg/mL) was added to the co-culture. In the supernatant in the IFN-γ + group, higher levels of IL-17A and IL-22but lower levels of IL-10 were found (Figure 5b). Besides, more IL-17 and RORγt but fewer Foxp3 were detected using Western blot (Figure 5c). These results indicated that IFN-γ mediated the differentiation inhibition of Th17 cells.

## 4 Discussion

Organ transplantation is the most effective method for the treatment of patients with end-stage organ failure. However, the therapeutic effect is affected by the possible rejection after the operation and the possible complications of lifelong use of immunosuppressants. Because of their immunosuppressive effect, MSCs have excellent potential in inducing graft immune tolerance to inhibit rejection and reduce the dosage of immunosuppressants. In the study of the ischemia-reperfusion renal transplantation model in rats, it was found that MSCs could reduce the chemotaxis of antigen-presenting cells (APCs), thus effectively reducing the inflammatory reaction in the process of transplantation [13]. Thus, the role of MSCs in renal transplantation has been confirmed. Studies by Miceli et al. [14] have shown that MSCs can reduce the inflammatory response caused by ischemia during renal transplantation, which is beneficial to graft survival. Mohr et al. [15] also showed that infusion of MSCs could significantly improve the decrease of superoxide dismutase (SOD) and glutathione peroxidase (GSH-Px) activity caused by ischemia-reperfusion, which is beneficial to the improvement of acute renal failure caused by ischemia-reperfusion.

Treg was first found to inhibit immune response and induce immune tolerance in the 1970s [16]. Natural regulatory T cell (Treg) accounts for about 5–10% of CD4^+^ T cells, expressing both CD4 and CD25 [17], and specifically expressing Foxp3 [17]. Treg can inhibit the activation and proliferation of CD4^+^ and CD8^+^ T lymphocytes, which is related to the direct contact between cells and paracrine cytokines IL-10 and TGF-β [18]. Interleukin-2 (IL-2) and other immunoregulatory molecules have been shown to control the generation of Tregs [19]. It can also indirectly inhibit the immune response by inhibiting the activity of APC while maintaining the microenvironment of immune tolerance. The most significant advantage of Treg in transplantation immunity is that it can actively transplant tolerance, that is, the Treg of the recipient who had reached immune tolerance to organ transplantation can be transferred to other recipients, and the recipient can also achieve the effect of immune tolerance. Sayitoglu et al. [20] confirmed this in an experiment on a mouse skin graft model and found that rejection would occur again after the removal of Treg. Studies of Park [19] and Tahvildari [21] also confirmed that transplantation of Treg could induce immune tolerance to MHC-mismatched heart or skin grafts. Thomann et al. [22] used donor-derived Treg in the islet transplantation model, which also significantly prolonged the survival time of the graft.

TGF-β is a crucial regulator of the immune response, regulating the occurrence and termination of immune response by regulating lymphocyte differentiation and apoptosis. A large number of studies have revealed the mechanism of this regulation. TGF-β can inhibit T lymphocyte proliferation mediated by IL-2 through the Smad3 pathway and directly inhibit T lymphocyte proliferation by downregulating cyclin D2, cyclin E, and c-myc [23,24]; upregulate the expression of FoxP3 and induce the differentiation of Treg. Given the immunosuppressive effect of TGF-β, its application in transplantation immunity has been widely studied. The study of mouse liver transplantation also confirmed a close relationship between TGF-β and liver regeneration [25]. In renal transplantation, TGF-β can significantly inhibit the inflammatory response in the early stage of acute inhibition of rejection, thus achieving the inhibition of chronic rejection [26]. Also, increasing the expression of islet B cell TGF-β in the type 1 diabetic mouse model is helpful to improve the condition of diabetes, which may be related to the increase of T lymphocyte apoptosis [27].

As a well-known immune enhancement factor, IFN-γ is the first discovered cytokine, a necessary condition for stimulating Th1 differentiation [28,29]. At the same time, it can also upregulate the expression of cellular MHC-I molecules, thus improving the ability of immune surveillance *in vivo* [30]. It can also mediate the activation of macrophages, enhance the lethality of macrophages and NK cells, promote the proliferation of B lymphocytes and the production of antibodies, and upregulate chemokines to promote the chemotaxis of inflammatory cells. According to the report, a large amount of IFN-γ can be detected in the supernatant of the co-culture system of CD4^+^ T cells and MSCs. Liu et al. believe that this phenomenon is caused by the high expression of TGF-β1 in MSCs [31]. In this study, the IFN-γ-neutralizing antibody significantly blocked the effects of MSCs on T cells, indicating that IFN-γ mediated the differentiation inhibition of Th17.

## 5 Conclusion

The present study demonstrated that TGF-β1/MSCs inhibited T lymphocyte proliferation and T lymphocyte immune status regulation. Moreover, TGF-β1/MSCs promoted the generation of Treg cells and suppressed the differentiation of Th17 cells. Besides, IFN-γ mediated the differentiation inhibition of Th17.
